# Perinatal Depression and Risk of Suicidal Behavior

**DOI:** 10.1001/jamanetworkopen.2023.50897

**Published:** 2024-01-09

**Authors:** Hang Yu, Qing Shen, Emma Bränn, Yihui Yang, Anna Sara Oberg, Unnur Anna Valdimarsdóttir, Donghao Lu

**Affiliations:** 1 Unit of Integrative Epidemiology, Institute of Environmental Medicine, Karolinska Institutet, Stockholm, Sweden; 2 Clinical Research Center for Mental Disorders, Shanghai Pudong New Area Mental Health Center, Tongji University School of Medicine, Shanghai, China; 3 Institute for Advanced Study, Tongji University, Shanghai, China; 4 Department of Medical Epidemiology and Biostatistics, Karolinska Institutet, Stockholm, Sweden; 5 Center of Public Health Sciences, Faculty of Medicine, University of Iceland, Reykjavík, Iceland

## Abstract

**Importance:**

Suicidal ideation is common among women with perinatal depression (PND). However, prospective data are limited on the risk, particularly long-term risk, of suicidal behavior (suicide attempt and completed suicide) among women with perinatal depression.

**Objective:**

To examine the association between PND and risk of short- and long-term suicidal behavior.

**Design, Setting, and Participants:**

A nationwide population-matched cohort study was conducted in Sweden including 86 551 women with PND from 2001 to 2017 and 865 510 unaffected women individually matched on age and calendar year at delivery. Sibling comparison was used to account for familial confounding. Data were analyzed from January 2022 to November 2023.

**Exposure:**

PND was identified through depression diagnosis or filled prescriptions of antidepressants from pregnancy to 1 year post partum in registers.

**Main Outcomes and Measures:**

All women were followed up for the first event of suicidal behavior recorded in registers. Hazard ratios (HR) of suicidal behavior were estimated using time-to-event analysis.

**Results:**

Women with PND (86 551 participants) received a diagnosis at a mean (SD) age of 30.67 (5.23) years. During a median (IQR) follow-up of 6.91 (3.62-10.88) years, 3604 events of suicidal behavior (incidence rate [IR], 5.62 per 1000 person-years) were identified among women with PND and 6445 (IR, 1.01 per 1000 person-years) among population-unaffected women. Women with PND had an elevated risk of suicidal behavior when compared with matched unaffected women (HR, 3.15; 95% CI, 2.97-3.35). Comparable, albeit somewhat attenuated, associations were yielded when comparing PND women with their PND-free sisters (HR, 2.75; 95% CI, 2.10-3.61). In the population-matched cohort, the association was greater for postnatal depression and among women without a history of psychiatric disorders. The excess risk was pronounced during the first year after diagnosis (HR, 7.20; 95% CI, 6.07-8.54), yet remained statistically significant during 5 to 18 years of follow-up (HR, 2.34; 95% CI, 2.12-2.57).

**Conclusions and Relevance:**

In this nationwide cohort study, women with PND were at an increased risk of suicidal behavior, particularly within the first year after diagnosis with persistent risk elevations throughout the 18 years of follow-up, highlighting the need for vigilant clinical monitoring of this vulnerable group.

## Key Points

**Question:**

Are mothers with perinatal depression at risk for suicidal behavior?

**Findings:**

In this cohort study of 952 061 participants with a maximal follow-up of 18 years, mothers with clinically diagnosed perinatal depression had a 3 times higher risk of suicidal behavior compared with mothers without perinatal depression. The excess risk was particularly high for postnatal depression and during the first year after diagnosis, which remained elevated through 18 years later and in comparison with their sisters without perinatal depression.

**Meaning:**

These findings suggest that vigilant clinical monitoring and interventions are needed for this vulnerable population to prevent such devastating events.

## Teaser

This cohort study evaluates the association between perinatal depression and risk of short- and long-term suicidal behavior.

## Introduction

Maternal suicide is an alarming public health issue and the second most common cause of death during the postnatal period.^1^ In fact, 13% to 36% of maternal deaths are attributable to suicide,^2^ and the consequences are devastating to the newborn and the family.^3^ Maternal suicide is linked to a complex interplay of risk factors, including history of psychiatric disorders,^4^ socioeconomic disparities, and inadequate access to health care service.^5^ It is of paramount importance to identify high-risk populations for preventing maternal suicide and suicidal attempt.

The co-occurrence of suicidality and perinatal depression (PND) has been well-documented.^6,7^ In fact, suicidal ideation is part of the typical screening for PND.^8^ Emerging evidence^9^ suggests that those with PND are a heterogenous group. For instance, antenatal (during pregnancy) and postnatal (maximally within 1 year after delivery) depression may have different causes and symptom clusters.^9^ Existing literature^10,11,12^ suggests that suicidal ideation is common among women with antenatal depression, while data on risk of suicidal behavior, including attempted and completed suicide, are lacking. Studies^13,14^ have shown that 3% to 19% of women with postnatal depression endorsed suicidal ideation. However, only a few small studies^15,16,17^ have examined the risk of suicide attempt or completed suicide^17^ among women with postnatal depression. These studies had a fairly short follow-up with few occurrences of suicidal behavior, yielding imprecise results.^15,16,17^ Additionally, a previous study^18^ illustrated mothers tended to use more violent methods for suicide after childbirth compared with the nonperinatal period. Yet, the characteristic of PND-related suicide method selection is poorly studied. Moreover, both depression and suicidal behavior are influenced by familial factors (eg, shared genetic and early-life environmental factors).^19,20^ To our knowledge, no studies have adjusted for potential familial confounding in this context, which can be well-addressed by using sibling comparison.^21^ To aid suicide prevention among mothers, we examined the association between PND and suicidal behavior by leveraging the nationwide register data in Sweden for a population-based matched cohort followed by a sibling comparison.

## Methods

### Data Source

The National Medical Birth Register (MBR) collects data on virtually all births in Sweden, including visits to prenatal care, delivery, and neonatal care^22,23^ since 1973. The National Patient Register (NPR)^24^ contains records of all hospitalization diagnoses since 1987 and over 80% of hospital-based outpatient diagnoses since 2001. The Swedish Prescribed Drug Register^25^ records filled prescriptions from all pharmacies in Sweden since July 2005. The National Cause of Death Register (CDR)^26^ has information on all deaths and causes since 1952. The Multi-Generation Register^27^ includes largely complete information on parents. Data linkage was carried out through the unique personal identity number assigned for every resident in Sweden.^28^

### Study Population and Design

To better illustrate the temporal patterns of suicidal behavior risk, we conducted a matched cohort study. Briefly, we identified 1 803 987 pregnancies from 1 041 419 women who gave birth during 2001 to 2017 from the MBR. PND was defined as the first depression diagnosis recorded during pregnancy or filled prescriptions of antidepressant medication (antenatal depression) or within 1 year after delivery (postnatal depression; identification codes are listed in eTable 1 in Supplement 1), as described elsewhere.^29^ After necessary exclusions (87 620 individuals) (eMethods in Supplement 1), 1 716 367 pregnancies from 1 029 215 women were identified as the study base and 86 551 women had an incident PND diagnosis.

Using incidence density sampling, women with PND (86 551 individuals) were 1:10 matched on age and year of delivery to women (865 510 individuals) who were free of PND at the same gestational age (for antenatal depression) or postnatal day (for postnatal depression). The matching date was the first-ever diagnosis date for women with PND and the corresponding gestational or postnatal day for matched unaffected women. Women were followed up from the matching date until first event of suicidal behavior, death, emigration, or December 31, 2018, whichever occurred first (eFigure 1 in Supplement 1). In addition, the follow-up of unaffected women was censored if they later received a diagnosis of PND.

The study was approved by the Swedish Ethical Review Authority, Sweden (2018/1515-31). Informed consent is waived for register-based studies according to Swedish laws. This study followed the Strengthening the Reporting of Observational Studies in Epidemiology (STROBE) reporting guideline.

### Ascertainment of Suicidal Behavior

We identified the first event of suicidal behavior, including both suicidal attempt and complete suicide, from the NPR and the CDR using *International Classification of Diseases, 10th Revision (ICD-10)* codes for both primary and secondary diagnoses and causes (eTable 1 in Supplement 1). The positive predictive value has been estimated as 100% for suicide recorded in the CDR^30^ and 94.8% for injury including suicidal attempt in the NPR.^31^ We also identified most common methods of suicidal behaviors (eTable 1 in Supplement 1)^32^ for the analysis of suicide method-specific risk.

### Covariates

Information on demographics (maternal age, calendar year at delivery, country of birth, and cohabitation status) was obtained from the MBR, while socioeconomic status (family annual household income and educational level) was acquired from the Longitudinal Integration Database for Health Insurance and Labor Market^33^ using the nearest 5-year recording to the time of matching. Pregnancy characteristics, including parity, smoking 3 months before pregnancy, and body mass index (BMI) during early pregnancy were obtained from the MBR as potential confounders.^34,35^ Hypertensive and diabetic diseases (potential confounders)^36,37,38^ were identified from the MBR and NPR, while history of suicidal behavior and/or psychiatric disorders^34,35^ were defined as any event or diagnosis recorded before the matching date in the NPR (*ICD-8, 9, *and *10* codes are listed in eTable 1 in Supplement 1). Pregnancy outcomes, including mode of delivery, gestational length, birth weight, and loss of offspring (including stillbirth and infant death within 1 year) are associated with postnatal depression and suicidal behavior.^17,34,39^ We collected information on these variables from the MBR and for loss of offspring additionally from the CDR.

### Statistical Analysis

We calculated crude incidence rates of suicidal behavior using the number of events divided by person-time at risk during follow-up. In the population-matched cohort, we estimated hazard ratios (HR) with 95% CIs of suicidal behavior by comparing women with PND to unaffected women using Cox regression models stratified by matching set (age and year at delivery). The underlying timescale was time since matching. To shed light on the temporal pattern, we divided the follow-up into 3 periods (<1 year, 1 to <5 years, and ≥5 years) and estimated period-specific HRs. We used flexible parametric survival models to estimate time-varying HRs^40^ for finer temporality.

We developed several models. In model 1, maternal age and calendar year at delivery were adjusted by stratifying on the matching set in the population-matched cohort. In model 2, demographic and socioeconomic factors were additionally included as covariates, and in model 3, we further included pregnancy characteristics. In model 4, the analysis of postnatal depression was additionally adjusted for pregnancy outcomes. Model 3 was considered the fully adjusted model with respect to potential confounders.

Since history of psychiatric disorders represents a potential risk factor for suicidal behavior,^41^ we conducted a stratified analysis (eMethods in Supplement 1) by history of psychiatric disorders (depression, other disorders, or no history) to evaluate potential risk modification. Similarly, we conducted further stratified analysis by history of suicidal behavior, age, calendar year at delivery, hypertensive diseases, and diabetes. The analysis of postnatal depression was additionally stratified by gestational age and birth weight groups.

The timing of PND diagnosis may reflect different disease subtypes.^42^ We therefore examined the association with PND diagnosed at different time windows. To inform potential prevention strategies in the future, we also assessed the suicide method-specific risk among most common suicide methods (>1%).

To test the robustness of our results, we carried out a series of additional analyses. First, to address familial confounding, we performed a sibling comparison^43,44^ that contrasted the rate of suicidal behavior among PND women (20 495 individuals) with their PND-free full sisters (23 433 individuals) by sibling set (eMethods in Supplement 1). Second, since antidepressants can be prescribed for mental disorders other than depression, we restricted the analysis to concern only PND identified through diagnoses. Third, consistent with prior studies,^45^ we included undetermined intention in defining suicidal behavior. We therefore limited the analysis to events recorded as intentional only (eMethods in Supplement 1). Last, we restricted analysis to suicidal behavior that resulted in hospitalization to understand the association with the most severe nonfatal outcome.

The data were prepared in SAS statistical software version 9.4 (SAS Institute), while statistical analyses were performed in R software, version 4.0.5 (R Project for Statistical Computing). A *P* value below .05 in a 2-sided test was considered statistically significant. Data were analyzed from January 2022 to November 2023.

## Results

### Characteristics

Of the 86 551 women with PND included in the study, 47 642 were affected in the antenatal period and 38 909 in the postnatal period. The mean (SD) age at PND diagnosis was 30.67 (5.23) years. Compared with matched unaffected women, women with PND were more likely to be born in Sweden, live alone, and have lower levels of education and income (eTable 2 in Supplement 1). Moreover, they were more likely to be primiparous, smoke 3 months before pregnancy, have a greater BMI during early pregnancy, and have a history of psychiatric disorders or suicidal behavior. In addition, they were more likely to experience cesarean delivery and loss of offspring within 1 year of birth.

### Risk of Suicidal Behavior

During a follow-up of up to 18 (median [IQR], 6.91 [3.62-10.88]) years (length of follow-up is summarized in eTable 3 in Supplement 1), we observed 3604 (IR, 5.62 per 1000 person-years) and 6445 (IR, 1.01 per 1000 person-years) events of suicidal behavior in women with PND and matched unaffected women, respectively.

Compared with matched unaffected women, women with PND had 3 times higher risk of suicidal behavior (fully-adjusted HR, 3.15; 95% CI, 2.97-3.35) (Table 1 and other models in eTable 4 in Supplement 1). Notably, the association was greater among women without a history of psychiatric disorders (HR, 3.63; 95% CI, 3.36-3.92) and comparable between women with history of depression (HR, 2.52; 95% CI, 2.14-2.96) and women with history of other psychiatric disorders (HR, 2.47; 95% CI, 2.15-2.84).

**Table 1.** Hazard Ratios (HRs) of Suicidal Behavior Among Women With Perinatal Depression (PND), Compared With Matched Unaffected Women

Characteristic	Throughout follow-up		<1 y		1 to <5 y		≥5 y	
	Suicidal behavior events, No. (IR/1000 PY)	HR (95% CI)^a^	Suicidal behavior events, No. (IR/1000 PY)	HR (95% CI)^a^	Suicidal behavior events, No. (IR/1000 PY)	HR (95% CI)^a^	Suicidal behavior events, No. (IR/1000 PY)	HR (95% CI)^a^
No PND	6445 (1.01)	1 [Reference]	546 (0.65)	1 [Reference]	2804 (1.01)	1 [Reference]	3095 (1.11)	1.00
PND	3604 (5.62)	3.15 (2.97-3.35)	667 (7.82)	7.20 (6.07-8.54)	1646 (5.85)	3.18 (2.90-3.49)	1291 (4.72)	2.34 (2.12-2.57)
By history of psychiatric disorders								
Without								
No PND	5052 (0.84)	1 [Reference]	400 (0.51)	1 [Reference]	2132 (0.83)	1 [Reference]	2520 (0.95)	1 [Reference]
PND	1442 (3.63)	3.63 (3.36-3.92)	248 (4.95)	8.85 (7.05-11.11)	609 (3.62)	3.80 (3.36-4.29)	585 (3.27)	2.81 (2.50-3.16)
With depression								
No PND	532 (4.49)	1 [Reference]	49 (2.32)	1 [Reference]	251 (4.28)	1 [Reference]	232 (6.00)	1 [Reference]
PND	1138 (9.77)	2.52 (2.14-2.96)	205 (11.96)	6.97 (4.24-11.45)	571 (10.38)	2.43 (1.91-3.10)	362 (8.17)	1.39 (1.07-1.81)
With other disorders								
No PND	861 (3.06)	1 [Reference]	97 (2.13)	1 [Reference]	421 (3.10)	1 [Reference]	343 (3.42)	1 [Reference]
PND	1024 (8.05)	2.47 (2.15-2.84)	214 (11.90)	4.24 (2.91-6.18)	466 (7.97)	2.43 (1.98-2.99)	344 (6.78)	1.82 (1.45-2.29)
*P* for interaction^b^	NA	<.001	NA	.007	NA	<.001	NA	<.001

Abbreviations: IR, Incidence rate; NA, not applicable; PY, person-years.

^a^ HRs were adjusted for maternal age, calendar year at delivery, educational level, annual household income, country of birth, cohabitation status, parity and body mass index during early pregnancy, smoking 3 months before pregnancy, diabetic and hypertensive disorders, history of psychiatric disorders, and history of suicidal behavior.

^b^ An interaction term was included in the Cox regression models and *P* for interaction as examined by Wald test.

The excess risk was greatest within 1 year following PND diagnosis (HR, 7.20; 95% CI, 6.07-8.54) (Table 1). Although declining over time, the risk remained doubled 5 or more years later (HR, 2.34; 95% CI, 2.12-2.57). Similar yet more precise temporal patterns are illustrated in the Figure.

**Figure.**
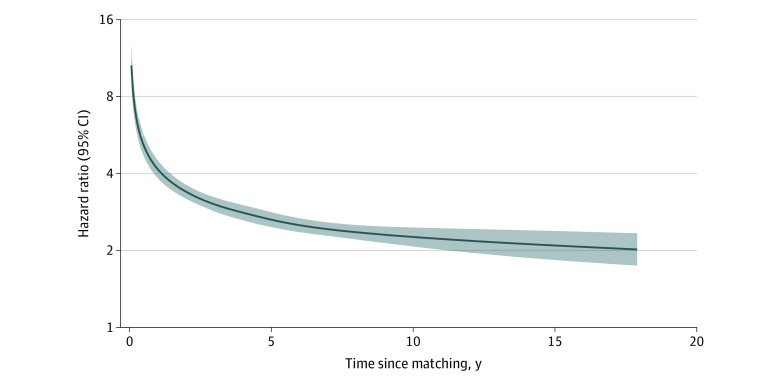
Hazard Ratios of Suicidal Behavior Among Women With Perinatal Depression Compared With Matched Unaffected Women Time-varying hazard ratios (solid line) and 95% CIs (shaded area) were derived from flexible parametric survival models allowing relative risk to vary over time. A spline with 5 degrees of freedom was used for the baseline hazard, and one with 3 degrees of freedom was used for the time-varying effect. Models were adjusted for maternal age, calendar year at delivery, educational level, annual household income, country of birth, cohabitation status, parity, body mass index, smoking 3 months before pregnancy, history of psychiatric disorders, history of suicidal behavior, and hypertensive and diabetic disorders.

Women with antenatal and postnatal depression were both at an increased risk of suicidal behavior throughout follow-up, although the associations were greater for postnatal depression (HR, 2.83; 95% CI, 2.61-3.07 for antenatal depression, and HR, 3.55; 95% CI, 3.26-3.86 for postnatal depression) (Table 2). The temporal patterns for both PND subtypes were similar to that found for PND overall. When stratifying on psychiatric history, greater associations were noted among women without psychiatric history for both PND subtypes (Table 2; eFigure 2 in Supplement 1). When adjusting for pregnancy outcomes, an even greater association was observed for postnatal depression (eTable 5 in Supplement 1).

**Table 2.** Associations of Antenatal and Postnatal Depression With Risk of Suicidal Behavior

Characteristic	Throughout follow-up		<1 y		1 to <5 y		>5 y	
	Suicidal behavior events, No. (IR/1000 PY)	HR (95% CI)^a^	Suicidal behavior events, No. (IR/1000 PY)	HR (95% CI)^a^	Suicidal behavior events, No. (IR/1000 PY)	HR (95% CI)^a^	Suicidal behavior events, No. (IR/1000 PY)	HR (95% CI)^a^
No PND	6445 (1.01)	1 [Reference]	546 (0.65)	1 [Reference]	2804 (1.01)	1 [Reference]	3095 (1.11)	1 [Reference]
Antenatal depression	1941 (5.14)	2.83 (2.61-3.07)	230 (4.86)	4.26 (3.26-5.57)	902 (5.58)	2.96 (2.61-3.36)	809 (4.80)	2.29 (2.03-2.58)
Postnatal depression	1663 (6.33)	3.55 (3.26-3.86)	437 (11.52)	9.74 (7.88-12.05)	744 (6.21)	3.42 (3.01-3.89)	482 (4.59)	2.41 (2.09-2.78)
By history of psychiatric disorders								
Without								
No PND	5052 (0.84)	1 [Reference]	400 (0.51)	1 [Reference]	2132 (0.83)	1 [Reference]	2520 (0.95)	1 [Reference]
Antenatal depression	655 (3.02)	3.14 (2.80-3.51)	54 (2.17)	4.24 (2.80-6.40)	280 (3.22)	3.65 (3.05-4.37)	321 (3.06)	2.64 (2.26-3.09)
Postnatal depression	787 (4.36)	4.14 (3.72-4.61)	194 (7.67)	12.31 (9.33-16.25)	329 (4.05)	3.91 (3.31-4.61)	264 (3.57)	3.02 (2.53-3.60)
By history of psychiatric disorders type								
With depression								
No PND	532 (4.49)	1 [Reference]	49 (2.32)	1 [Reference]	251 (4.28)	1 [Reference]	232 (6.00)	1 [Reference]
Antenatal depression	769 (8.60)	2.31 (1.93-2.76)	103 (8.11)	5.72 (3.21-10.20)	395 (9.38)	2.14 (1.64-2.79)	271 (7.84)	1.41 (1.05-1.88)
Postnatal depression	369 (13.64)	2.94 (2.31-3.75)	102 (23.02)	9.00 (4.58-17.66)	176 (13.68)	3.20 (2.20-4.66)	91 (9.34)	1.33 (0.88-2.00)
With other than depression								
No PND	861 (3.06)	1 [Reference]	97 (2.13)	1 [Reference]	421 (3.10)	1 [Reference]	343 (3.42)	1 [Reference]
Antenatal depression	517 (7.19)	2.39 (2.01-2.84)	73 (7.47)	2.69 (1.62-4.48)	227 (6.92)	2.43 (1.86-3.17)	217 (7.40)	1.94 (1.48-2.54)
Postnatal depression	507 (9.17)	2.52 (2.11-3.02)	141 (17.19)	5.77 (3.62-9.19)	239 (9.31)	2.46 (1.88-3.20)	127 (5.94)	1.65 (1.19-2.28)
*P* for interaction^b^	NA	<.001	NA	<.001	NA	<.001	NA	<.001

Abbreviations: HR, hazard ratio; IR, incidence rate; NA, not applicable; PND, perinatal depression; PY, person-years.

^a^ HRs were adjusted for age, calendar year at delivery, educational level, annual household income, country of birth, cohabitation status, parity and body mass index during early pregnancy, smoking 3 months before pregnancy, diabetic and hypertensive disorders, history of psychiatric disorders, and history of suicidal behavior.

^b^ An interaction term was included in the Cox regression models and *P* for interaction as examined by Wald test.

### Suicidal Behavior Methods

The most common suicidal behavior methods among women with PND were poisoning throughout follow-up and across different time windows (Table 3). However, among the common methods, the association was found most pronounced for hanging (HR, 7.29; 95% CI, 4.42-12.03) (Table 3). Of note, within 1 year after PND, the risk increase was highest for suicidal poisoning (HR, 11.76; 95% CI, 9.86-14.02). We found statistically comparable associations throughout follow-up, except for suicidal behavior by poisoning, for which the association was greater for postnatal depression (eTable 6 in Supplement 1).

**Table 3.** Associations of Perinatal Depression (PND) and Risk of Suicidal Behavior by Different Methods

Characteristic and method	Throughout follow-up		<1 y		1 to <5 y		>5 y	
	Suicidal behavior events, No. (IR/1000 PY)	HR (95% CI)^a^	Suicidal behavior events, No. (IR/1000 PY)	HR (95% CI)^a^	Suicidal behavior events, No. (IR/1000 PY)	HR (95% CI)^a^	Suicidal behavior events, No. (IR/1000 PY)	HR (95% CI)^a^
Poisoning								
No PND	3367 (0.53)	1 [Reference]	229 (0.27)	1 [Reference]	1494 (0.54)	1 [Reference]	1644 (0.59)	1 [Reference]
PND	2615 (4.08)	4.39 (4.06-4.73)	493 (5.78)	13.49 (10.50-17.33)	1204 (4.28)	4.52 (4.01-5.09)	918 (3.35)	3.16 (2.80-3.56)
Cutting or piercing								
No PND	752 (0.12)	1 [Reference]	65 (0.08)	1 [Reference]	355 (0.13)	1 [Reference]	332 (0.12)	1 [Reference]
PND	374 (0.58)	3.09 (2.58-3.68)	68 (0.80)	8.32 (4.43-15.64)	181 (0.64)	2.94 (2.25-3.83)	125 (0.46)	2.47 (1.83-3.33)
Falling								
No PND	167 (0.03)	1 [Reference]	20 (0.02)	1 [Reference]	70 (0.03)	1 [Reference]	77 (0.03)	1 [Reference]
PND	55 (0.09)	3.07 (1.99-4.73)	18 (0.21)	13.81 (3.04-62.82)	21 (0.07)	3.62 (1.70-7.71)	16 (0.06)	1.85 (0.86-3.98)
Hanging								
No PND	79 (0.01)	1 [Reference]	8 (0.009)	1 [Reference]	28 (0.01)	1 [Reference]	43 (0.02)	1 [Reference]
PND	81 (0.13)	7.29 (4.42-12.03)	18 (0.21)	NA	23 (0.08)	3.44 (1.19-9.92)	40 (0.15)	11.70 (5.26-26.01)

Abbreviations: HR, hazard ratio; IR, incidence rate; NA, not applicable; PY, person-years.

^a^ HRs were adjusted for age, calendar year at delivery, educational level, annual household income, country of birth, cohabitation status, parity and body mass index during early pregnancy, smoking 3 months before pregnancy, diabetic and hypertensive disorders, history of psychiatric disorders, and history of suicidal behavior.

### Additional Analyses

When comparing full sisters with and without PND, the association between PND and suicidal behavior was somewhat attenuated but PND-affected women remained at substantially elevated risk (HR, 2.75; 95% CI, 2.10-3.61) (Table 4), particularly for those without history of psychiatric disorders (HR, 2.94; 95% CI, 2.09-4.13). Comparable associations were found between prenatal and postpartum depression (eTable 7 in Supplement 1).

**Table 4.** Hazard Ratios (HRs) of Suicidal Behavior Among Women With Perinatal Depression (PND), Compared With Unaffected Full Siblings

Characteristic	Suicidal behavior events, No.	IR/1000 PY	HR (95% CI)		
			Model 1^a^	Model 2^b^	Model 3^c^
Throughout follow-up					
No PND	261	1.24	1 [Reference]	1 [Reference]	1 [Reference]
PND	741	4.83	3.55 (2.85-4.42)	3.35 (2.65-4.22)	2.75 (2.10-3.61)
By history of psychiatric disorders					
Without					
No PND	193	1.00	1 [Reference]	1 [Reference]	1 [Reference]
PND	321	3.12	3.02 (2.23-4.10)	2.94 (2.13-4.04)	2.94 (2.09-4.13)
With					
No PND	68	4.11	1 [Reference]	1 [Reference]	1 [Reference]
PND	420	8.29	2.39 (1.49-3.84)	2.29 (1.40-3.75)	2.40 (1.38-4.18)
*P* for interaction^d^	NA	NA	.44	.43	.57

Abbreviations: IR, incidence rate; NA, not applicable; PY, person-years.

^a^ Maternal age and calendar year at delivery (ie, the matching factors) were inherently adjusted for in the population-matched cohort and were adjusted for in the sibling cohort.

^b^ Demographic characteristics including educational level, annual household income, country of birth, and cohabitation status were additionally adjusted for.

^c^ Pregnancy characteristics, including body mass index during early pregnancy, smoking 3 months before pregnancy, parity, diabetic and hypertensive disorders, history of psychiatric disorders, and history of suicidal behavior were additionally adjusted for.

^d^ An interaction term was included in the Cox regression models and *P* for interaction as examined by Wald test.

In stratified analyses, we found a greater association among women without a history of suicidal behavior (HR, 3.27; 95% CI, 3.07-3.49 vs HR, 2.47; 95% CI, 2.08-2.93 in women with a history of suicidal behavior; *P* for interaction = .003) (eTable 8 in Supplement 1). The association was more pronounced among women who gave a birth during 2010 to 2017 or between 26 to 30 years of age. The associations were not modified by pregnancy complications (eTable 8 in Supplement 1), gestational age, or birth weight of the newborns (eTable 9 in Supplement 1).

Similar associations were noted for antenatal depression diagnosed before and after 14 weeks of gestation (eTable 10 in Supplement 1), whereas a greater association was found for postnatal depression diagnosed within 6 months postnatal compared with diagnosis at 7 to 12 months. Finally, comparable results were observed when using only clinically diagnosed PND, suicidal behavior events coded as intentional, or suicidal behavior resulting in hospitalization (eTable 11 in Supplement 1).

## Discussion

In this nationwide matched cohort study of 952 061 women with up to 18 years of follow-up, we found that women with a clinical diagnosis of PND have an elevated risk of suicidal behavior compared with population-matched women or their full sisters without PND. Attenuated yet still substantially elevated risks were observed when comparing with full sisters without PND who share partial genetic and familial environmental factors with affected women. Importantly, such excess risk was apparent among women regardless of their history of psychiatric disorders, suggesting that PND is linked to an added risk of suicidal behavior beyond the risk associated with psychiatric disorders occurring before the perinatal period. Moreover, the risk elevations were particularly high shortly after the PND diagnosis, and despite the rapid decline over time, remained throughout 18 years of follow-up.

A few studies have reported that perinatal psychiatric disorders,^46^ mostly postnatal ones,^1,7,15^ are associated with a higher risk of suicidal behavior. Although depression is the most common type of postnatal psychiatric disorder, few studies have specifically addressed postnatal depression and found a positive association with suicidal ideation and behavior.^17,47^ In line with previous studies, our study shows that postnatal depression is associated with an increased risk of suicidal behavior in both population and sibling comparisons. For antenatal depression, evidence on the risk of suicidal behavior is scarce, despite several cross-sectional studies indicating a positive correlation between antenatal depression and suicidal ideation.^6,10,11,12^ To our knowledge, our study is the first to illustrate that women with antenatal depression are at elevated risk of suicidal behavior compared with women without PND in a cohort study.

History of psychiatric disorders is a risk factor for both PND and suicidal behavior,^46,48^ serving as a potential confounder in the studied association. Therefore, history of psychiatric disorders has been well accounted for throughout our analyses. We found that the association was greater in women without than with history of psychiatric disorders, suggesting psychiatric history cannot explain our findings and a greater association with PND in this group. Where previous studies often excluded PND with a prior major depression or other psychiatric disorders,^16,17^ we rather demonstrated that even among women with a psychiatric history, PND was associated with increased risk of suicidal behavior. This emphasizes the pressing need to include PND women with psychiatric history in future PND studies. Regarding the lesser association noted among women with psychiatric history, they tend to discontinue the use of psychotropic medication during pregnancy and post partum^49,50^; it is plausible that women with both PND and a prior psychiatric disorder are more likely to receive active treatment during the perinatal period, resulting in a lesser association with suicidal behavior. Future studies are needed to understand how treatment during the perinatal period may modify such risk.

It is not surprising that the risk of suicidal behavior peaked right after PND diagnosis, given the ongoing episode or symptoms at diagnosis. This finding highlights the urgent need to actively monitor suicidality among women who recently received a diagnosis of PND. Importantly, the long-term impact of PND on suicidal behavior has been neglected in the literature and often limited to only 1 year postnatal follow-up.^15,16,17,47,51^ We reported that the risk elevation of suicidal behavior, although attenuated over time, remained elevated even 18 years later. Emerging data indicate that some PND could last for 7 years after delivery^52^ and may entail nonperinatal mental disorders.^53^ Such consequences may explain the risk of suicidal behavior later in life. Some risk factors (eg, low social support^54^) may extend beyond the perinatal period and be associated with increased suicidal behavior in the long run. Future research is needed to understand the driving forces of the long-term increased risk following PND.

Availability and social acceptability have been identified as 2 key factors influencing the choice of suicide method.^55^ Compared with men, women are more likely to use nonviolent methods of suicide, such as poisoning, hanging, and so forth.^56^ We found that the most common suicidal behavior method among women with PND was poisoning. Given that these women are often prescribed antidepressants, health care clinicians should be alert to potential misuse of medications.^57^ Although less common, the most pronounced association was found for suicidal behavior committed by hanging.^58^ As case fatality following hanging is high (70%), strategies focusing on providing a safe environment should be considered for prevention purposes.^59^

In our recent study on PND-associated with mortality risk, we found that women with PND had more than 6 times higher risk of suicide than women without PND.^29^ However, the absolute risk for suicide is small (218 completed suicide out of 10 049 suicidal behavior events) and the results in the present study largely represent the risk of suicide attempt.

### Strengths and Limitations

The major strengths of our study are the nationwide coverage with prospectively collected and complete follow-up data and the advanced analytic approaches (eg, sibling comparison and flexible parametric survival models). The large sample size enabled detailed subgroup analyses and to assess common suicidal behavior methods.

However, there are some limitations to be noted. First, we could not capture PND diagnosed in primary care unless it led to the filling of prescribed antidepressants. However, as these women were classified into the reference group, this leads to attenuated associations. Second, there might be an underestimate of suicidal behavior. A previous study indicates nearly 50% of suicide attempters in Sweden seek no medical treatment.^60^ Nevertheless, similar results were yielded by restricting to suicidal behavior resulting in hospitalizations, which are completely captured in registers, largely minimizing the impact of misclassification or surveillance bias, if any. Third, although we have comprehensively adjusted for confounders, including those shared by siblings, our data did not capture domestic violence,^61^ alcohol consumption,^62^ and birth trauma (only applicable to postnatal depression).^63^ However, our finding appears robust as an unmeasured confounder would need to have a relative risk of 6.56 with both PND and suicidal behaviors to fully account for the observed result.^64^ Additionally, Sweden is a high-income country with universal health care. Our findings may not be generalized to countries with different socioeconomic profiles and health care accessibility. As the majority of the study population is White women, future studies with sufficient racial and ethnic diversity are warranted.

Our findings have important implications for clinicians. First, maternal care clinicians typically prioritize the physical health of the pregnant woman and the fetus. Our study on antenatal depression–associated risk of suicidal behavior emphasizes the importance of focusing on women and their mental well-being during pregnancy. Second, the sustained risk over 18 years necessitates a paradigm shift in how we should approach PND, advocating for extended monitoring and support beyond the perinatal period. Most importantly, the striking risk elevation of suicidal behavior within 1 year after PND reinforces the necessity of strategies that effectively detect early signs and act in a timely manner for suicide prevention.

## Conclusions

Our findings suggest that women with clinically diagnosed PND are at an increased risk of suicidal behavior, particularly within 1 year after PND, yet throughout 18 years of follow-up. This highlights the pressing need for vigilant clinical monitoring and prompt intervention for this vulnerable population to prevent such devastating outcomes, regardless of prepregnancy history of psychiatric disorders.

## Notes

Supplement 1. **eTable 1.** Identification Codes for Perinatal Depression, Classification of Common Causes of Deaths and Codes for Identifying Hypertensive and Diabetic Disorders
eMethods
**eFigure 1.** Flowchart **eTable 2.** Characteristics of Women With Perinatal Depression (PND) and Without PND **eFigure 2.** Hazard Ratios (HRs) of Suicidal Behavior Among Women With Antenatal and Postnatal Depression, Compared With Matched Unaffected Women **eTable 3.** Length of Follow-Up by Different Censoring Points **eTable 4.** Hazard Ratios (HRs) With 95% CIs of Suicide Behavior Among Women With Perinatal Depression, Compared With Their Matched Unaffected Individuals: Models 1 and 2 **eTable 5.** Hazard Ratios (HRs) of Suicide Behavior Among Women With Postnatal Depression, With Additional Adjustment for Pregnancy Outcomes **eTable 6.** Hazard Ratios (HRs) of Suicidal Behavior by Methods Among Women With Antenatal and Postnatal Depression Separately, Compared With Matched Unaffected Women **eTable 7.** Hazard Ratios (HRs) With 95% CIs of Suicidal Behavior Among Women With Antenatal and Postnatal Depression, Compared With Unaffected Full Siblings **eTable 8.** Hazard Ratios of Suicidal Behavior Among Women With Perinatal Depression Stratified by History of Suicidal Behavior, Age, Calendar Year at Delivery and Pregnancy Complications **eTable 9.** Hazard Ratios of Suicidal Behavior Among Women With Postnatal Depression Stratified by Gestational Age and Birth Weight **eTable 10.** Hazard Ratios (HRs) of Suicidal Behavior Among Women With Antenatal and Postnatal Depression, by Time at Diagnosis **eTable 11.** Hazard Ratios (HRs) of Suicidal Behavior Among Women With Perinatal Depression (PND): Sensitivity Analyses Using Different Ascertainment of PND and Suicidal Behavior

Supplement 2.
Data Sharing Statement
