# Supplementary material for: Perinatal Depression and Risk of Suicidal Behavior
Source: JAMA Netw Open. 2024 Jan 9;7(1):e2350897. doi: 10.1001/jamanetworkopen.2023.50897 (PMC10777256; doi:10.1001/jamanetworkopen.2023.50897)
Supplement: Supplement 1. — eTable 1. Identification Codes for Perinatal Depression, Classification of Common Causes of Deaths and Codes for Identifying Hypertensive and Diabetic Disorders eMethods eFigure 1. Flowchart eTable 2. Characteristics of Women With Perinatal Depression (PND) and Without PND eFigure 2. Hazard Ratios (HRs) of Suicidal Behavior Among Women With Antenatal and Postnatal Depression, Compared With Matched Unaffected Women eTable 3. Length of Follow-Up by Different Censoring Points eTable 4. Hazard Ratios (HRs) With 95% CIs of Suicide Behavior Among Women With Perinatal Depression, Compared With Their Matched Unaffected Individuals: Models 1 and 2 eTable 5. Hazard Ratios (HRs) of Suicide Behavior Among Women With Postnatal Depression, With Additional Adjustment for Pregnancy Outcomes eTable 6. Hazard Ratios (HRs) of Suicidal Behavior by Methods Among Women With Antenatal and Postnatal Depression Separately, Compared With Matched Unaffected Women eTable 7. Hazard Ratios (HRs) With 95% CIs of Suicidal Behavior Among Women With Antenatal and Postnatal Depression, Compared With Unaffected Full Siblings eTable 8. Hazard Ratios of Suicidal Behavior Among Women With Perinatal Depression Stratified by History of Suicidal Behavior, Age, Calendar Year at Delivery and Pregnancy Complications eTable 9. Hazard Ratios of Suicidal Behavior Among Women With Postnatal Depression Stratified by Gestational Age and Birth Weight eTable 10. Hazard Ratios (HRs) of Suicidal Behavior Among Women With Antenatal and Postnatal Depression, by Time at Diagnosis eTable 11. Hazard Ratios (HRs) of Suicidal Behavior Among Women With Perinatal Depression (PND): Sensitivity Analyses Using Different Ascertainment of PND and Suicidal Behavior [file jamanetwopen-e2350897-s001.pdf]

Supplemental Online Content

Yu H, Shen Q, Bränn E, et al. Perinatal depression and risk of suicidal behavior. *JAMA Netw Open*. 2023;7(1):e2350897. doi:10.1001/jamanetworkopen.2023.50897

eTable 1. Identification Codes for Perinatal Depression, Classification of Common Causes of Deaths and Codes for Identifying Hypertensive and Diabetic Disorders
eMethods
eFigure 1. Flowchart
eTable 2. Characteristics of WOMEN WITH PERINATAL DEPRESSION (PND) AND WITHout PND
eFigure 2. Hazard Ratios (HRs) of Suicidal Behavior Among Women With Antenatal and Postnatal Depression, Compared With Matched Unaffected Women
eTable 3. Length of Follow-Up by Different Censoring Points
eTable 4. Hazard Ratios (HRs) With 95% CIs of Suicide Behavior Among Women With Perinatal Depression, Compared With Their Matched Unaffected Individuals1: Models 1 and 2
eTable 5. Hazard Ratios (HRs) of Suicide Behavior Among Women With Postnatal Depression, With Additional Adjustment for Pregnancy Outcomes
eTable 6. Hazard Ratios (HRs) of Suicidal Behavior by Methods Among Women With Antenatal and Postnatal Depression Separately, Compared With Matched Unaffected Women
eTable 7. Hazard Ratios (HRs) With 95% CIs of Suicidal Behavior Among Women With Antenatal and Postnatal Depression, Compared With Unaffected Full Siblings
eTable 8. Hazard Ratios of Suicidal Behavior Among Women With Perinatal Depression Stratified by History of Suicidal Behavior, Age, Calendar Year at Delivery and Pregnancy Complications
eTable 9. Hazard Ratios of Suicidal Behavior Among Women With Postnatal Depression Stratified by Gestational Age and Birth Weight
eTable 10. Hazard Ratios (HRs) of Suicidal Behavior Among Women With Antenatal and Postnatal Depression, by Time at Diagnosis
eTable 11. Hazard Ratios (HRs) of Suicidal Behavior Among Women With Perinatal Depression (PND): Sensitivity Analyses Using Different Ascertainment of PND and Suicidal Behavior

This supplemental material has been provided by the authors to give readers additional information about their work.

© 2024 Yu H et al. *JAMA Network Open.*

eTable 1. Identification Codes for Perinatal Depression, Classification of Common Causes of Deaths and Codes for Identifying Hypertensive and Diabetic Disorders

	**ICD 10**		
**Perinatal depression**			
Diagnoses from the Patient Register or MBR	F32.0, F32.1, F32.2, F32.3, F32.8, F32.9, F33.0, F33.1, F33.2, F33.3, F33.4, F33.8, F33.9 and F53.0		
	**ATC**		
Antidepressants from the Drug Register or MBR prescription	N06A		
	**ICD-10**		
**Suicidal behavior**			
Suicidal attempt and complete suicide	X60-X84, Y10-Y34		
- Poison	X60-X69, Y10-Y19		
- Cutting or piercing	X78, X79, Y28, Y29		
- Falling	X80, X81, Y30, Y31		
- Hanging	X70, Y20		
- Drowning	X71, Y21		
- Transport	X82, Y32		
- Fire	X76, Y26		
- Other	X72-75, X77, X83-X84, Y22-25, Y27, Y33-Y34		
	**ICD 8**	**ICD 9**	**ICD 10**
**Calendar year**	1968-1986	1987-1996	1997-
**History of psychiatric disorders**			
- Depression	-*	296,298,300,311	F32-F33
- Other psychiatric disorders	-*	291,292,295,297, 299,301-311	F10-F31, F34-F69
**Hypertensive disorder**			
- Pregestational	401-405	642C-642H	O10-O11, I10-I15
- Hypertension	-	642D-642G, 642X	O12, O16
**Diabetic disorder**			
- Pregestational	250	648A	E10-E14, O241-O243
- Gestational	-	648W	O244

Abbreviation: ATC, The Anatomical Therapeutic Chemical; ICD, International Classification of Diseases; MBR, Medical Birth Register.

* ICD 8 was not used as psychiatric diagnosis is well covered in the registers only after 1987.

© 2024 Yu H et al. *JAMA Network Open.*

eMethods

Exclusion criteria and definition of perinatal depression in the study population

Among the 1,803,987 pregnancies identified from the Medical Birth Register, we excluded 678 pregnancies without information on gestational age, 51,806 pregnancies with multiples, 35,133 pregnancies from women already affected by perinatal depression (PND) in a prior pregnancy, and 3 erroneous records (death recorded before pregnancy), in total 87, 620 pregnancies were excluded.

We defined PND as any depression diagnosis recorded during pregnancy until one year postpartum. The start of pregnancy, defined as date of last menstrual period, was estimated from date of delivery and gestational age, which is primarily estimated based on an ultrasound assessment performed before 18 gestational weeks.[28]

Stratified analysis

We performed stratified analyses by history of psychiatric disorders, history of suicidal behavior, age, calendar year at delivery, hypertensive diseases, and diabetes; and for postnatal depression, we performed such analyses by gestational age and birth weight groups. For each stratified analysis, we added an interaction term between PND and the stratification factor and tested the statistical significance of the interaction term using Wald test to indicate the P for interaction.

Sibling cohort study design

To address potential familial confounding, 821,540 women with at least one sibling were identified utilizing family linkage in The Multi-generation Register (MGR). All women were followed from first recorded pregnancy until emigration, death, or December 31, 2018 whichever occurred first. We then identified PND women who had at least one full and parous sister without PND recorded in the MBR. To be consistent with the population design, we randomly assigned one PND-free pregnancy from each sister for comparison. In the sibling analysis we included 20,495 PND (11,011 antenatal depression and 9,484 postnatal depression) women and 23,433 unaffected full sisters (eFigure1).

Additional analysis

Third, in line with previous studies our definition of suicidal behavior included events coded as “undetermined intention. We performed an analysis restricting to events recorded as “intentional” only.

© 2024 Yu H et al. *JAMA Network Open.*

eFigure 1. Flowchart

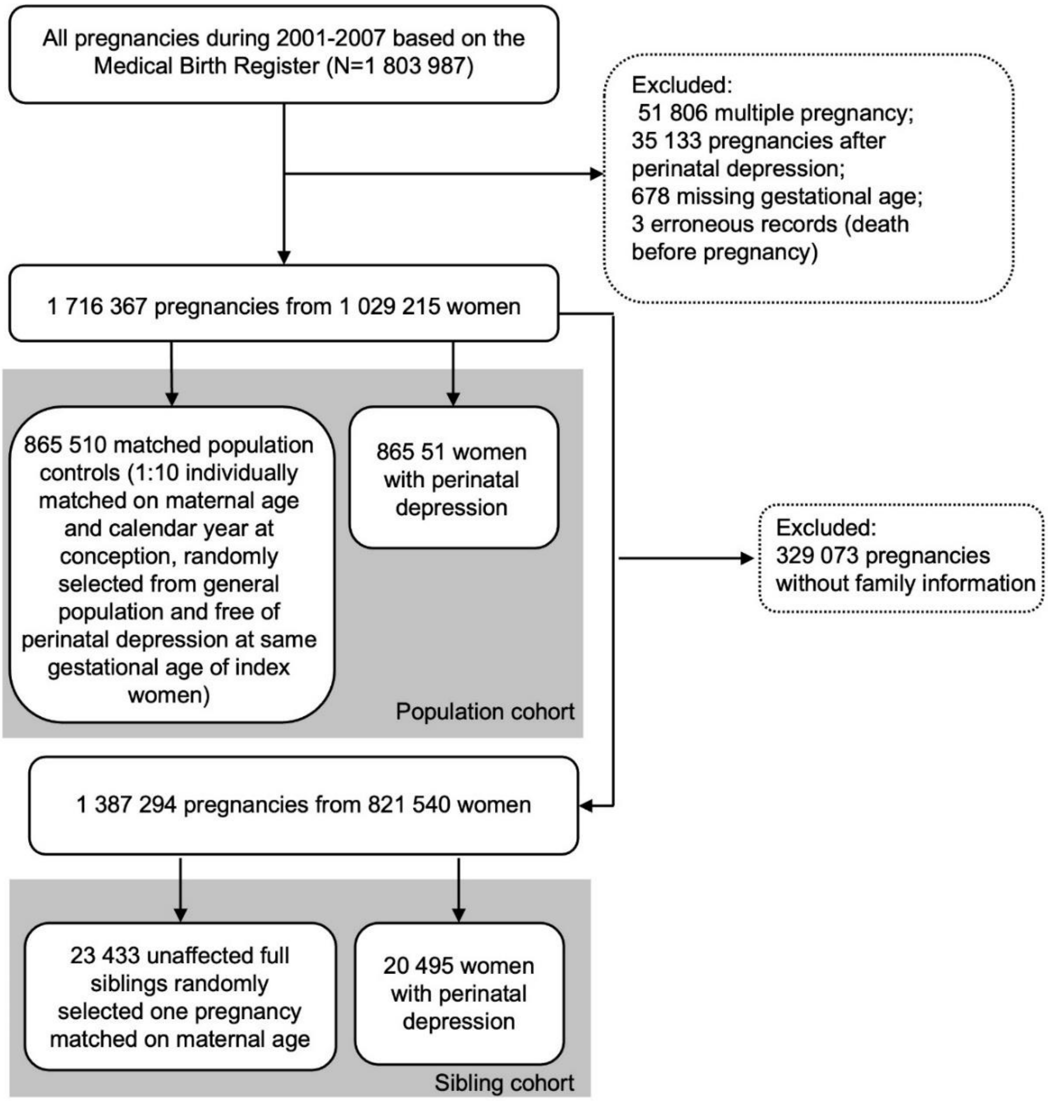

eTable 2. Characteristics of WOMEN WITH PERINATAL DEPRESSION (PND) AND WITHout PND, N (%).

	No PND (N=865,510)	PND (N=86551)
Demographic characteristics		
**Age at delivery, years**		
<u><</u>20	23,197 (2.7)	2,309 (2.7)
21-25	136,241 (15.7)	13,687 (15.8)
26-30	261,016 (30.2)	26,032 (30.1)
31-35	268,111 (31.0)	26,760 (30.9)
36-40	144,412 (16.7)	14,480 (16.7)
<u>></u>41	32,533 (3.8)	3,283 (3.8)
**Calendar year at delivery**		

© 2024 Yu H et al. *JAMA Network Open.*

2001-2005	120,294 (13.9)	11,916 (13.8)
2006-2010	267,734 (30.9)	26,911 (31.1)
2011-2015	325,780 (37.6)	32,616 (37.7)
2016-2017	151,702 (17.5)	15,108 (17.5)
**Country of birth**		
Sweden	650,114 (75.1)	73,295 (84.7)
Other/Unknown	215,396 (24.9)	13,256 (15.3)
**Cohabitation status**		
Cohabitation	806,763 (93.2)	78,860 (91.1)
Single	16,147 (1.9)	3,433 (4.0)
Unknown	42,600 (4.9)	4,258 (4.9)
**Educational level**		
Primary school	103,395 (11.9)	13,924 (16.1)
High school	324,665 (37.5)	36,211 (41.8)
College and beyond	419,393 (48.5)	35,701 (41.2)
Unknown	18,057 (2.1)	715 (0.8)
**Household income**		
Q1	205,599 (23.8)	27,840 (32.2)
Q2	211,544 (24.4)	21,773 (25.2)
Q3	213,757 (24.7)	19,504 (22.5)
Q4	216,553 (25.0)	16,719 (19.3)
Unknown	18,057 (2.1)	715 (0.8)
Pregnancy characteristics		
**Smoking before pregnancy**		
No	767,753 (88.7)	70,463 (81.4)
1-9 cig./day	40,236 (4.6)	8,192 (9.5)
≥10 cig./day	11,181 (1.3)	3,310 (3.8)
Unknow	46,340 (5.4)	4,586 (5.3)
**BMI during early pregnancy, kg/m^2**		
<18.5	20,863 (2.4)	2,137 (2.5)
18.5-25	479,033 (55.3)	43,416 (50.2)
25-30	199,661 (23.1)	20,817 (24.1)
≥30	99,020 (11.4)	13,179 (15.2)
Unknown	66,933 (7.7)	7,002 (8.1)
**Hypertensive diseases**		
No	836,644 (96.7)	82,660 (95.5)
Gestational hypertension or preeclampsia	11,604 (1.3)	1,622 (1.9)
Pregestational hypertension	17,262 (2.0)	2,269 (2.6)
**Diabetes**		
No	838,050 (96.8)	82,847 (95.7)
Gestational diabetes	11,872 (1.4)	1,654 (1.9)
Pregestational diabetes	15,588 (1.8)	2,050 (2.4)
**History of psychiatry disorders**		

© 2024 Yu H et al. *JAMA Network Open.*

No	793,566 (91.7)	50,862 (58.8)
Depression	23,735 (2.7)	17,411 (20.1)
Other psychiatric disorders	48,209 (5.6)	18,278 (21.1)
**History of suicidal behavior**		
No	847,518 (97.9)	79,441 (91.8)
Yes	17,992 (2.1)	7,110 (8.2)
**Parity**		
1	390,564 (45.1)	45,429 (52.5)
2	313,133 (36.2)	25,023 (28.9)
3	112,643 (13.0)	11,069 (12.8)
≥4	49,170 (5.7)	5030 (5.8)
Pregnancy outcomes		
**Mode of delivery**		
Cesarean section	142,374 (16.4)	19,410 (22.4)
Vaginal, assisted	60,365 (7.0)	6,480 (7.5)
Vaginal, not assisted	662,771 (76.6)	60,661 (70.1)
**Gestational age, weeks**		
<37	40,633 (4.7)	6,128 (7.1)
≥37	824,877 (95.3)	80,423 (92.9)
**Birth weight, g**		
2500-4199	746,174 (86.2)	74,027 (85.4)
≥4200	90,864 (10.5)	8,347 (9.6)
Unknown	1,210 (0.1)	173 (0.2)
**Loss of offspring**		
No	861,379 (99.5)	85,686 (99.0)
Stillbirth	2,951 (0.3)	619 (0.7)
Infant death (0-365 days)	1,180 (0.1)	246 (0.3)

Abbreviation: BMI, body mass index (kg/m2); N, number; %, percentage; Q, quartile; cig, cigarette.

© 2024 Yu H et al. *JAMA Network Open.*

eFigure 2. Hazard Ratios (HRs) of Suicidal Behavior Among Women With Antenatal and Postnatal Depression, Compared With Matched Unaffected Women

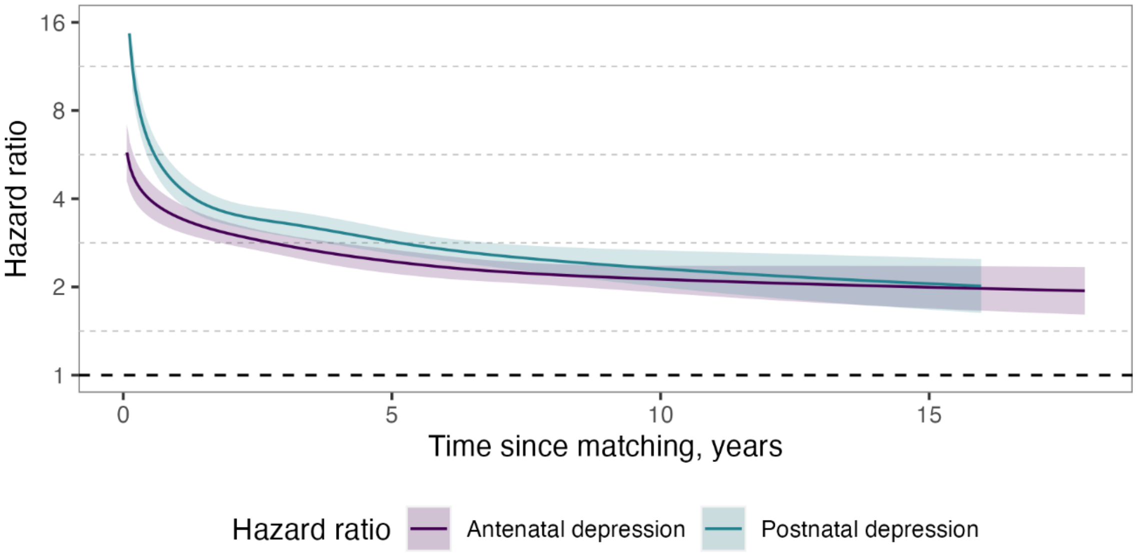

Time-varying hazard ratios and 95% confidence intervals were derived from flexible parametric survival models allowing relative risk to vary over time. A spline with 5 df was used for the baseline hazard, and 3 df was used for the time-varying effect. Models were adjusted for maternal age, calendar year at delivery, educational level, annual household income, country of birth, cohabitation status, parity and body mass index during early pregnancy, smoking before pregnancy, history of psychiatric disorders, history of suicidal behavior, and hypertensive and diabetic disorders.

© 2024 Yu H et al. *JAMA Network Open.*

eTable 3 Length of Follow-Up by Different Censoring Points

Category	Min. (Years)	Q1 (Years)	Median (Years)	Mean (Years)	Q3 (Years)	Max. (Years)
If censoring at:						
Emigration	0.003	1.92	3.64	4.25	6.01	17.30
Death	0.003	3.36	6.47	6.83	9.89	17.91
Suicidal behavior	0.003	2.22	4.38	5.12	7.35	17.89
End of study period	0.014	3.94	7.21	7.68	11.08	18.67

© 2024 Yu H et al. *JAMA Network Open.*

eTable 4 Hazard Ratios (HRs) With 95% CIs of Suicide Behavior Among Women With Perinatal Depression, Compared With Their Matched Unaffected Individuals1: Models 1 and 2

	Throughout follow-up		<1 year		1 to <5 years		≥5 years	
	HR (95%CI) [a]	HR (95%CI) [b]	HR (95%CI) [a]	HR (95%CI) [b]	HR (95%CI) [a]	HR (95%CI) [b]	HR (95%CI) [a]	HR (95%CI) [b]
No PND	1.00	1.00	1.00	1.00	1.00	1.00	1.00	1.00
PND	5.37 (5.10-5.64)	4.71 (4.47-4.96)	11.08 (9.61-12.79)	10.15 (8.74-11.79)	5.73 (5.32-6.19)	5.01 (4.63-5.42)	4.04 (3.73-4.38)	3.53 (3.25-3.84)
**By *history of psychiatric disorders***								
Without								
No PND	1.00	1.00	1.00	1.00	1.00	1.00	1.00	1.00
PND	4.16 (3.86-4.48)	3.89 (3.61-4.20)	9.34 (7.54-11.57)	9.20 (7.38-11.48)	4.40 (3.92-4.94)	4.14 (3.68-4.66)	3.27 (2.92-3.65)	3.01 (2.68-3.37)
With								
No PND	1.00	1.00	1.00	1.00	1.00	1.00	1.00	1.00
PND	2.73 (2.48-3.01)	2.62 (2.38-2.89)	5.23 (3.99-6.84)	5.13 (3.90-6.75)	2.63 (2.29-3.03)	2.51 (2.18-2.89)	1.82 (1.55-2.13)	1.74 (1.48-2.04)
$P_{interaction}$	<0.001	<0.001	0.002	0.002	<0.001	<0.001	<0.001	<0.001

Abbreviations: HR, hazard ratio; CI, confidence interval; PND, perinatal depression.

[a] Estimates were inherently adjusted for matching factors (age and calendar year at delivery) by stratifying on the matching set.

[b] Estimates were additionally adjusted for country of birth, cohabitation status, education level, and household income.

[c] An interaction term was included in the Cox regression models and P for interaction as examined by Wald test.

© 2024 Yu H et al. *JAMA Network Open.*

eTable 5. Hazard Ratios (HRs) of Suicide Behavior Among Women With Postnatal Depression, With Additional Adjustment for Pregnancy Outcomes

	**Throughout follow-up**	**<1 year**	**1 to <5 years**	**≥5 years**
	**HR (95%CI)** [a]	**HR (95%CI)** [a]	**HR (95%CI)** [a]	**HR (95%CI)** [a]
No PND	1.00	1.00	1.00	1.00
Postnatal depression	3.52 (3.24-3.82)	9.55 (7.71-11.83)	3.38 (2.98-3.84)	2.40 (2.07-2.77)

Abbreviations: HR, hazard ratio; CI, confidence interval; PND, perinatal depression.

[a] Estimates were inherently adjusted for matching factors (age and calendar year at delivery) by stratifying on the matching set, country of birth, cohabitation status, education level, and household income, parity, BMI at early pregnancy, smoking before pregnancy, hypertensive disease, diabetes, history of psychiatric disorders, history of suicidal behavior, the mode of delivery, gestational age, birth weight, and offspring death.

© 2024 Yu H et al. *JAMA Network Open.*

eTable 6. Hazard Ratios (HRs) of Suicidal Behavior by Methods Among Women With Antenatal and Postnatal Depression Separately, Compared With Matched Unaffected Women

	Throughout follow-up		0-1 year		1 to <5 years		≥5 years	
	N (IR [a])	HR (95% CI) [b]	N (IR [a])	HR (95% CI) [b]	N (IR [a])	HR (95% CI) b	N (IR [a])	HR (95% CI) b
Poisoning								
Antenatal depression	1,397 (3.70)	3.53 (3.17-3.92)	159 (3.36)	6.50 (4.34-9.74)	643 (3.98)	3.67 (3.1-4.34)	595 (3.53)	2.91 (2.49-3.39)
Postnatal depression	1,218 (4.63)	4.75 (4.26-5.3)	334 (8.81)	18.01 (13.14-24.68)	561 (4.68)	4.70 (3.98-5.56)	323 (3.08)	2.89 (2.39-3.50)
Cutting or piercing								
Antenatal depression	222 (0.59)	2.68 (2.10-3.41)	26 (0.55)	2.70 (1.08-6.77)	111 (0.69)	2.73 (1.88-3.94)	85 (0.50)	2.24 (1.53-3.30)
Postnatal depression	152 (0.58)	3.05 (2.35-3.95)	42 (1.11)	20.53 (8.22-51.3)	70 (0.58)	2.46 (1.67-3.62)	40 (0.38)	2.35 (1.47-3.75)
Hanging								
Antenatal depression	42 (0.11)	8.69 (4.16-18.15)	<5	-	16 (0.10)	5.97 (1.31-27.22)	24 (0.14)	13.17 (4.55-38.11)
Postnatal depression	39 (0.15)	4.70 (2.42-9.12)	16 (0.42)	-	7 (0.06)	0.82 (0.16-4.35)	16 (0.15)	7.06 (2.14-23.24)
Falling								
Antenatal depression	28 (0.07)	3.15 (1.71-5.83)	7 (0.15)	61.63 (2.34-1620.76)	11 (0.07)	4.79 (1.56-14.78)	10 (0.06)	1.84 (0.68-4.99)
Postnatal depression	27 (0.10)	2.89 (1.62-5.15)	11 (0.29)	8.71 (1.59-47.81)	10 (0.08)	2.96 (1.17-7.51)	6 (0.06)	1.56 (0.44-5.55)

Abbreviations: N, number of suicidal behavior events; IR, Incidence rate; HR, hazard ratio; CI, confidence interval; PND, perinatal depression.

[a] Per 1000 person-years, unadjusted.

[b] HRs were adjusted for age, calendar year at delivery, educational level, annual household income, country of birth, cohabitation status, parity and BMI during early pregnancy, smoking 3 months before pregnancy, diabetic and hypertensive disorders, history of psychiatric disorders, and history of suicidal behavior.

Note: Due to a small number of events, we only analyzed common suicide methods by PDN subtypes.

© 2024 Yu H et al. *JAMA Network Open.*

eTable 7. Hazard Ratios (HRs) With 95% CIs of Suicidal Behavior Among Women With Antenatal and Postnatal Depression, Compared With Unaffected Full Siblings

	N (IR) 1000 Pys	Model 1[a] HR (95% CI)	Model 2[b] HR (95% CI)	Model 3[c] HR (95% CI)
**Throughout follow-up**				
No PND	261 (1.24)	1.00	1.00	1.00
Antenatal depression	362 (4.09)	3.19 (2.4-4.24)	2.91 (2.14-3.95)	1.97 (1.37-2.83)
Postnatal depression	379 (5.83)	4.01 (2.95-5.45)	3.88 (2.81-5.37)	3.70 (2.58-5.30)
**By *history of psychiatric disorders***				
Without history of psychiatric disorders				
No PND	193 (1)	1.00	1.00	1.00
Antenatal depression	123 (2.32)	3.09 (2.04-4.68)	2.83 (1.74-4.59)	3.08 (1.79-5.29)
Postnatal depression	198 (3.97)	2.46 (1.78-3.41)	2.10 (1.46-3.02)	2.31 (1.55-3.43)
$P_{interaction}{}^{d}$		0.533	0.713	0.59
With history of depression				
No PND	30 (5.09)	1.00	1.00	1.00
Antenatal depression	148 (7.14)	1.90 (0.99-3.64)	1.83 (0.84-3.96)	2.25 (0.99-5.10)
Postnatal depression	95 (14.45)	3.10 (1.32-7.25)	3.13 (1.19-8.26)	3.73 (1.30-10.64)
$P_{interaction}{}^{d}$		0.153	0.224	0.186
with history of other psychiatric disorders				
No PND	38 (3.56)	1.00	1.00	1.00
Antenatal depression	91 (6.11)	1.44 (0.73-2.82)	1.22 (0.54-2.77)	1.11 (0.46-2.69)
Postnatal depression	86 (10.15)	2.74 (1.27-5.93)	2.47 (0.99-6.16)	3.16 (1.19-8.38)
$P_{interaction}{}^{d}$		0.88	0.743	0.635

Abbreviations: N, number of suicidal behavior; HR, hazard ratio; CI, confidence interval; Pys, person-years; IR, Incidence rate; PND, perinatal depression.

[a] Maternal age and calendar year at delivery (i.e., the matching factors) were inherently adjusted for in the population- matched cohort; and were adjusted for in the sibling cohort.

[b] Demographic characteristics including educational level, annual household income, country of birth and cohabitation status were additional adjusted for.

© 2024 Yu H et al. *JAMA Network Open.*

[c] Pregnancy characteristics including BMI during early pregnancy, smoking 3 months before pregnancy, parity, diabetic and hypertensive disorders, history of psychiatric disorders and history of suicidal behavior were additionally adjusted for.

[d] An interaction term was included in the Cox regression models and P for interaction as examined by Wald test.

© 2024 Yu H et al. *JAMA Network Open.*

eTable 8. Hazard Ratios of Suicidal Behavior Among Women With Perinatal Depression Stratified by History of Suicidal Behavior, Age, Calendar Year at Delivery and Pregnancy Complications

	No PND		PND	
	N (IR [a])	HR	N (IR [a])	HR (95%CI) [b]
History of suicidal behavior				
No	5,707 (0.90)	1.00	2,561 (4.29)	3.27 (3.07-3.49)
Yes	738 (7.44)	1.00	1,043 (23.50)	2.47 (2.08-2.93)
$P_{interaction}{}^{c}$				0.003
Age				
≤20	742 (4.12)	1.00	302 (17.58)	2.17 (1.81-2.60)
21-25	1,807 (1.82)	1.00	941 (9.57)	2.81 (2.52-3.14)
26-30	1,604 (0.85)	1.00	1,057 (5.61)	3.83 (3.43-4.27)
31-35	1,365 (0.68)	1.00	826 (4.10)	3.47 (3.07-3.92)
36-40	737 (0.68)	1.00	391 (3.54)	3.17 (2.67-3.76)
≥41	190 (0.78)	1.00	87 (3.49)	2.93 (2.06-4.16)
$P_{interaction}{}^{c}$				<0.001
Calender year at delivery				
2001-2009	4,389 (1.12)	1.00	2,307 (5.97)	2.95 (2.74-3.17)
2010-2017	2,056 (0.82)	1.00	1,297 (5.10)	3.60 (3.27-3.98)
$P_{interaction}{}^{c}$				0.001
Hypertensive diseases				
No	6,153 (0.99)	1.00	3,408 (5.57)	3.19 (3.00-3.39)
Gestational hypertension or preeclampsia	85 (1.02)	1.00	83 (7.00)	3.30 (2.08-5.22)
Pregestational hypertension	207 (1.63)	1.00	113 (6.61)	2.38 (1.61-3.51)
$P_{interaction}{}^{c}$				0.34
Diabetes				
No	6,165 (0.99)	1.00	3,410 (5.56)	3.19 (3.00-3.39)
Gestational diabetes	92 (1.07)	1.00	85 (6.98)	3.17 (2.03-4.96)
Pregestational diabetes	188 (1.66)	1.00	109 (7.09)	2.48 (1.65-3.72)
$P_{interaction}{}^{c}$				0.48

Abbreviations: N, number of suicidal behavior; IR, Incidence rate; HR, hazard ratio; CI, confidence interval; PND, perinatal depression.

[a] Per 1000 person-years, unadjusted.

[b] Estimates were inherently adjusted for matching factors (age and calendar year at delivery) by stratifying on the matching set, country of birth, cohabitation status, education level, and household income, parity, BMI at early pregnancy, smoking before pregnancy, hypertensive disease, diabetes, history of psychiatric disorders, and history of suicidal behavior.

[c] An interaction term was included in the Cox regression models and P for interaction as examined by Wald test

© 2024 Yu H et al. *JAMA Network Open.*

eTable 9. Hazard Ratios of Suicidal Behavior Among Women With Postnatal Depression Stratified by Gestational Age and Birth Weight

	No PND		Postnatal Depression	
	N (IR [a])	HR	N (IR [a])	HR
Gestational age, weeks				
<37	387 (1.27)	1.00	147 (8.40)	3.54 (2.56-4.89)
≥37	6,058 (0.99)	1.00	1516 (6.18)	3.49 (3.20-3.81)
$P_{interaction}$[c]				0.936
Birth weight, g				
<2500	268 (1.34)	1.00	95 (8.06)	2.48 (1.68-3.67)
≥2500	6,167 (0.99)	1.00	1562 (6.24)	3.54 (3.25-3.86)
$P_{interaction}$[c]				0.096

Abbreviations: N, number of suicidal behavior; IR, Incidence rate; HR, hazard ratio; CI, confidence interval; PND, perinatal depression.

[a] Per 1000 person-years, unadjusted.

[b] Estimates were inherently adjusted for matching factors (age and calendar year at delivery) by stratifying on the matching set, country of birth, cohabitation status, education level, and household income, parity, BMI at early pregnancy, smoking before pregnancy, hypertensive disease, diabetes, history of psychiatric disorders, and history of suicidal behavior.

[c] An interaction term was included in the Cox regression models and P for interaction as examined by Wald test.

eTable 10. Hazard Ratios (HRs) of Suicidal Behavior Among Women With Antenatal and Postnatal Depression, by Time at Diagnosis

	No of suicidal behavior[a] in unexposed/exposed individuals	Model 1[b] HR (95% CIs)	Model 2[c] HR (95% CIs)	Model 3[d] HR (95% CIs)
**Antenatal depression**				
Within 13 weeks	1,714 (0.91)/1,032 (5.35)	5.74 (5.22-6.31)	4.89 (4.42-5.41)	2.99 (2.64-3.39)
14 weeks onwards	1,916 (1.04)/909 (4.91)	4.79 (4.35-5.27)	4.18 (3.78-4.62)	2.70 (2.40-3.03)
**Postnatal depression**				
Within 6 months	1,348 (1.08)/808 (6.54)	5.90 (5.31-6.57)	5.31 (4.75-5.93)	4.08 (3.60-4.61)
7-12 months	1,467 (1.03)/855 (6.14)	5.17 (4.66-5.74)	4.62 (4.14-5.15)	3.18 (2.82-3.59)

Abbreviations: HR, hazard ratio; CI, confidence interval.

[a] Per 1000 person-years, unadjusted.

[b] Maternal age and calendar year at delivery (i.e., the matching factors) were inherently adjusted for in the population- matched cohort; and were controlled for in the sibling cohort.

[c] Demographic characteristics including educational level, annual household income, country of birth and cohabitation status were additional adjusted for.

[d] Pregnancy characteristics including BMI during early pregnancy, smoking before pregnancy, parity, diabetic and hypertensive disorders, history of psychiatric disorders and history of suicidal behavior were additionally adjusted for.

© 2024 Yu H et al. *JAMA Network Open.*

eTable 11. Hazard Ratios (HRs) of Suicidal Behavior Among Women With Perinatal Depression (PND): Sensitivity Analyses Using Different Ascertainment of PND and Suicidal Behavior

	No of suicidal bahaviors[a] in unexposed/exposed individuals	HR (95% CI)
**Restricting to PND ascertained from clinical diagnoses**		
Perinatal depression [b]	1,670 (1.03)/1313 (8.24)	4.60 (4.11-5.14)
**Restricting to suicidal behavior with clear intention**		
Perinatal depression [b]	3,577 (0.56)/2,848 (4.44)	4.22 (3.92-4.55)
**Restricting to suicidal behavior from hospitalization**		
Perinatal depression [b]	2,902 (0.45)/2,276 (3.55)	4.15 (3.81-4.51)

Abbreviations: HR, hazard ratio; CI, confidence interval.

[a] Per 1000 person-years, unadjusted.

[b] HRs were adjusted for maternal age, calendar year at delivery, educational level, annual household income, country of birth, cohabitation status, parity and BMI during early pregnancy, smoking before pregnancy, history of psychiatric disorders, diabetic and hypertensive disorders. HRs were additionally adjusted for maternal age and calendar year at delivery in sibling comparison.

© 2024 Yu H et al. *JAMA Network Open.*
