# Supplementary material for: Perinatal Depression and Risk of Suicidal Behavior
Source: JAMA Netw Open. 2024 Jan 9;7(1):e2350897. doi: 10.1001/jamanetworkopen.2023.50897 (PMC10777256; doi:10.1001/jamanetworkopen.2023.50897)
Supplement: Supplement 2. — Data Sharing Statement [file jamanetwopen-e2350897-s002.pdf]

Data Sharing Statement

Yu. Perinatal Depression and Risk of Suicidal Behavior. *JAMA Netw Open*. Published January 09, 2024. doi:10.1001/jamanetworkopen.2023.50897

Data
Data available: No

Additional Information
Explanation for why data not available: Due to privacy protection measures, such as the General Data Protection Regulation (GDPR), the registers' information is not publicly accessible. Access to Swedish register resources is granted only after an ethical evaluation by the relevant authorities. For further information on acquiring access to Swedish register data, consult the Swedish National Board of Health and Welfare's website (https://bestalladata.socialstyrelsen.se/, email: registerservice@socialstyrelsen.se) and/or the Statistics Sweden website (https://www.scb.se/vara-tjanster/bestall-data-och-statistik/, email: scb@scb.se)
